# Barriers and facilitators of dental service utilization by children aged 8 to 11 years in Enugu State, Nigeria

**DOI:** 10.1186/s12913-016-1341-6

**Published:** 2016-03-15

**Authors:** Nneka Kate Onyejaka, Morenike Oluwatoyin Folayan, Nkiruka Folaranmi

**Affiliations:** University of Nigeria Teaching Hospital, Enugu, Nigeria; Obafemi Awolowo University, Ile-Ife, Nigeria; University of Nigeria, Enugu, Nigeria

**Keywords:** Utilization, Oral health care, Barriers, Family structure

## Abstract

**Background:**

Multiple factors influence a child’s ability to access oral health care. The aim of this study was to identify factors that facilitated and served as barriers to children’s utilization of oral health care services in Enugu, Nigeria.

**Methods:**

The study recruited 1406 primary school pupils aged 8 to 11 years. All the children received oral health education, with the aid of an oral health education curriculum appropriate for their age. After this, referral letters were given to the children. Twelve months later, the study participants were revisited in their schools to obtain information on their reasons for utilizing, or not utilizing an oral health care service in the last 12 months. The association between socio-economic status; form of parenthood; number of siblings, birth rank and reasons for utilization and non-utilization of dental services were assessed. Influence on the child’s predisposition to oral health service utilization on dental visit was also assessed.

**Results:**

Only 116 (14.7 %) of the 791 children accessible during the 12 months follow-up visit had visited the dental clinic and the main reason for utilization was the desire to fulfill the dentist’s request for dental visit (41.9 %) while parents’ inability to make out time for a dental visit (43.3 %) was the main reason for non-utilization. The odds of utilizing oral health care services for study participants from the middle (AOR: 0.50; CI: 0.31–0.79; *P* = 0.003) and low (AOR: 0.24; CI: 0.13–0.45; *p* = <0.001) socioeconomic strata, and those living with guardians/relatives (AOR: 0.08; CI: 0.01–0.60; *p* = 0.01) were decreased when compared to those living with both parents, respectively. Respondents with positive perception about dental service utilization had increased odds of utilizing oral health care (AOR: 2.96; CI: 1.48–5.90; *p* = 0.002).

**Conclusion:**

Dentists can be strong motivators for children to utilize oral health care. Time is a significant barrier for the utilization of dental services. The programs designed to address barriers to oral health care utilization for children should be geared towards overcoming the possible threats that socio-economic status and type of parents they have may pose, to reduce inequity in dental service utilization.

## Background

Oral health is defined as the status of oral and related tissues that enable an individual to eat, speak and socialize without active disease, discomfort or embarrassment and also contributes to the general well being of the individual [1]. It is part of general health and should not be considered in isolation, as it contributes to the individual’s health related quality of life [2].

One way of maintaining oral health status is by making regular dental visits to health care centres to reinforce preventive oral health habits, and to ensure prompt diagnosis and management of dental anomalies. Utilization of health care services is measured by the number of visits to oral health care centres per year, or the number of people who made at least one visit in the previous year [3]. Generally, reports from sub-Saharan Africa show very low utilization of oral health care services and visits are mostly undertaken for symptomatic reasons [4, 5].

Studies in Nigeria also show similar findings among children, with socioeconomic status of the family [6], perception of needs [7, 8], low dental awareness and cost [9] being the major barriers to utilization of oral health care services. This is of concern as the level of untreated caries ranges from 77.2 to 98.6 % [10] despite the low mean dmft/DMFT reported in children [10, 11]. Also, a large number of children live with the sequelae of dental caries [12] resulting in low productivity and low quality of life for many of them [13]. It is therefore very important to identify ways to facilitate children’s utilization of oral health care services, for both preventive and curative care in Nigeria.

Previous studies showed that family related factors such as socio-economic status, type of parents, family size and birth rank influence access to, and utilization of oral health care services by children. A strong association between socioeconomic status and utilization of oral health care services has also been demonstrated in Chile [14], with family income having a significant negative correlation with dental visit [15]. Also in Chile and Brazil, children from low socio-economic backgrounds utilized oral healthcare services less frequently than those from high socio-economic background [16, 17].

Also, family structure is associated with self-reported dental attendance pattern [17]. In Britain and the United States, children of young single mothers with more than two children have poorer health outcomes due to inappropriate monitoring of oral health by the mothers [18, 19]. Also, children growing up with single mothers and step fathers were less likely to visit the dentist regularly compared with those in conventional nuclear families in Germany [20].

In Nigeria however, globalization and modernization have resulted in a modification of the extended family structure with families having fewer kinship network with distant relatives [21]. Very few studies have tried research on how family structure in Nigeria influences utilization of oral health services. A study conducted by Ola, et al. [22] established that children living with single mothers or children without a parent were unlikely to have visited the dentist, and the number of siblings and birth rank had no association with utilization of oral health services. This study for the first time, highlighted that family related factors are significant influencers of use of dental services by children in Nigeria, as identified in other developed countries.

This study aimed at determining the association between socio-economic status, type of family, form of parenthood, birth rank and number of siblings, and utilization of oral health care services by children issued with referral letters in Enugu, Nigeria. It also identified reasons parents gave as barriers and facilitators to utilization of oral health care services by their children in Enugu metropolis. Findings from the study will be used to plan interventions to improve utilization of oral health services in the study population.

## Methods

### Study area

The study was conducted in Enugu metropolis, Enugu State of Nigeria. Study participants were recruited from the Enugu East, Enugu North and Enugu South Local Government Areas (LGA) of the State where the population of school pupils aged 8 to 11 years was 41,853. In South-Eastern Nigeria, monogamy is the common type of marriage and the average birth per woman is 4.8 [23].

### Study design and study population

This was a cross sectional study with measures collected at two different time points 12 months apart. Pupils aged 8 to 11 years, schooling in the three Local Government Areas (LGAs) in Enugu metropolis were recruited for the study. Pupils with special needs (physically, medically and mentally compromised) and expected to regularly utilize oral health care services were excluded from the study.

### Sample size

Using the sample size formula by Araoye [24], based on a 15 % prevalence of oral health care service utilization by children in Lagos [7], a margin of error of 5 % and a confidence level of 95 %, the total sample size required to get a referred population of 200 pupils was 1,333.3 which was rounded up to 1,400 pupils. The sample was recruited from 30 schools.

### Sampling technique

A multistage stratified sampling technique was used to recruit study participants. The first stage involved selection of a proportionate representation of the schools per LGA through balloting, from a list of schools provided by the State Ministry of Education. Prior to this selection, schools in each LGA were stratified into public and private schools to ensure the selection of a proportionate representation between private and public primary schools in each LGA. The total number of schools selected by balloting was 12 public primary schools and 18 private primary schools.

The second stage involved the random selection of classes with a large population of children aged between 8 and 11 years. The class registration list which also showed the ages of the students was used to determine the classes with the highest number of pupils aged 8 to 11 years. All the classes with a high proportion of children in this age bracket, in each school were listed and two classes where students’ recruitment would take place in each of the 30 schools were randomly selected by balloting.

The third stage involved the selection of 47 pupils from the two classes in each school to participate in the study. All pupils within the stipulated age group were asked to pick a ballot paper which had either a ‘yes’ or a ‘no’ written on it. There were only 47 ‘yes’ responses and the pupils who picked them participated in the study.

### Data collection tool

During the first visit, information collected included age at last birthday, gender and socio-economic status. The socio-economic status of each child was calculated using multiple indices obtained from a scoring index, which combined the mother’s level of education and occupation of the father [25]. Father’s occupation was grouped into; professional (score 1); civil servants (score 2); unskilled, unemployed, civil servants with primary education (score 3) while mother’s level of education was categorized into tertiary education (score 0); secondary (score 1) and primary or no school education (score 2). Each child’s family social class was obtained by adding the score of the father’s occupation to the score of the mother’s level of education. A total score of 1 (class 1) was categorized as upper class, total score of 2 (class II) was upper middle class, total score of 3 (class III) middle class, total score of 4 (class IV) the lower middle class, and a total score of 5 (class V) was the lower class.

This social classification system has been used in Nigeria and found valid and reliable [26, 27]. For data analysis, social classes I and II were merged to become the high socioeconomic class, social class III was the middle socio-economic class while social class IV and V were merged to become the low socioeconomic class. Information was also collected on family structure. This included data on the type of family (monogamous, polygamous), type of parenting (living with parents, single parent, step mother or other relations), birth rank and number of siblings.

At the 12 month follow up visit, information on reasons why the child was able or not able to make a dental visit was collected using mainly closed ended questions. The information was extracted from the children first and confirmation or otherwise extracted from by the parents through phone interviews. Where there was disparity in the response between the two, the parent was informed about the response of the child and asked to corroborate it. The reason given for the visit or non-visit to the dental clinic by the parent was taken as the final response to the question. For those who made the dental visits, information on the dates was also generated.

To generate the information on reason for making dental visits, children and their parents were required to make a ‘yes’ or ‘no’ response to three questions. These were: ‘to fulfil the dentist’s request’, ‘to satisfy child/ward’s demand to visit the dentist’, ‘visit prompted by symptoms’ and ‘others’. They were also expected to make a ‘yes’ or ‘no’ response to six assertions exploring reasons for not making dental visits. They were: ‘parents/guardian had no time to visit the dentist’, ‘anticipated cost of treatment was expensive’, ‘my teeth are healthy’, ‘parents did not respond to prompting to make dental visits’, ‘lost referral letter’, ‘forgot to give referral letter to parents’. Children and parents were allowed to provide other reasons for making, or not making dental visits.

### Standardization of field workers

Three dentists were recruited as field workers and trained on the data collection procedure and details of the study collection tool. Discussions and clarifications were made about the content of the questionnaire during training and field testing.

### Study procedure

Ethical approval for the study was obtained from the University of Nigeria Health Research Ethics Committee (IRB 00002323). Permission was also obtained from school authorities in Enugu in addition to obtaining parental consent and child assent child participation in the study. Parents interested in participating in the study were asked to provide their physical addresses and telephone numbers for possible interviews about the child for such cases where the information was not be obtainable from the child or teacher. All practicing dentists in all registered dental clinics in Enugu metropolis were contacted, the aim and objectives of the study explained, and the role expected of them highlighted.

The questionnaires were filled for the study participants by trained field workers before the oral health education was conducted for the pupils, using the Oral Health Education training curriculum for children in primary schools by the Oral Wellness Study Group [28]. Information was collected on the pupils’ socio demographic profile and family structure and they were all given referral letters to visit any of the registered oral health centres within Enugu metropolis, listed in their referral letters. Children were encouraged to take the referral letters along with them to the clinics and drop them with the dentist there.

Twelve months after the school visit, the Principal Investigator re-visited the schools and obtained information from the study participants on their reasons for attending or not attending an oral health care service centre. The pupils were taken through another oral health education class on this second visit using the same oral health education curriculum.

### Data analysis

Assessment of the predisposition of study participants to dental visits was done through the development of a composite score derived from the history of dental visits made by them in the past- (visited the dentist in the past-1, never visited the dentist-0), regularity of past dental visits (regular once in a year visit −2; occasional visit-1; never visited a dentist-0); and reasons for past dental visits (curative-0; preventive-1). A maximum score of ‘4‘ indicated a very positive predisposition, a score of ‘3’ indicated positive predisposition, a score of ’2’ indicated negative predisposition, a score of ‘1’ indicated moderately negative predisposition, while the minimum score of ‘0’ indicated a very negative predisposition.

Statistical Package of Social Science (SPSS) version 15 was used for data analysis. Descriptive analysis was conducted to determine the proportion of children who visited a dental clinic pre and post intervention, reasons for visit(s) and non-visit(s) to a dental clinic post intervention, and the date of dental visit(s).

Bivariate analysis was conducted to test the association between the child’s socio-economic status, type of family, form of parenthood, birth rank, number of siblings and reasons for making visits and not making visits to a dental clinic. The association between the child’s predisposition to utilizing dental services and reasons for dental service utilization was also determined. For ease of analysis, predisposition was categorised into 2: negative (scores 0, 1 and 2) and positive (scores 3 and 4).

A model was developed to determine predictors of utilization and non-utilization of dental services. The model included factors that show an association between health service utilization and non-utilization with p values < 0.2 [29]. Study participants’ predisposition to dental service utilization was also included in the models. The level of statistical significance was inferred at *p* < 0.05.

## Result

### Socio-demographic profile of study participants

One thousand, four hundred and six (1406) pupils participated in the study. The mean age ± (SD) of the study participants was 9.32 ± (1.08) years. The mean age± (SD) for male participants was 9.42 ± (1.09) years and 9.23 ± (1.07) years for female participants. Table 1 shows the general characteristics of the study participants. The modal age of the study participants was eight (8) years (29.7 %). Most of the study participants were from monogamous family (95.4 %), and living with both parents (84.4 %). Also, 40.5 % of participants had three to four siblings.

**Table 1:** General characteristics of the study participants (*N* = 1406)

Variables	Frequency
	*n *(%)
Age (years)	
8	418(29.7)
9	372(26.5)
10	363(25.8)
11	253(18.0)
Sex	
Male	672(47.8)
Female	734(52.2)
Socio-economic status	
High	521(37.1)
Middle	439(31.2)
Low	446(31.7)
Type of family	
Monogamy	1341(95.4)
Polygamy	65(4.6)
Form of parenthood	
Both parents	1186(84.4)
One of the parents	46(3.2)
Relative/guardian	174(12.4)
Birth rank	
Only child	38(2.7)
First child	326(23.2)
Last child	313(22.3)
Others	729(51.8)
Number of siblings	
0	38(2.7)
1–2	245(17.4)
3–4	570(40.5)
>4	553(39.4)

Table 2 shows the socio-demographic profile of the 132 (9.4 %) study participants who had ever visited an oral health centre for dental care. Many (64.4 %) of them were from the high socio-economic stratum. There was a statistically significant difference in the number of study participants who had made past dental visits by socio-economic status (*P* <0.001).

**Table 2:** Distribution of study participants who had utilized oral health care services before referral by socio-economic status, family structure and distance (*N* = 1406)

Variables	Oral health care service utilization			
	*N* = 132	*N* = 1,274		
	Yes (*n* %)	No (*n* %)	Total (*n* %)	*P* value
Socio-economic status				<0.001
High	85(64.4)	436(34.2)	521(37.1)	
Middle	31(23.5)	408(32.0)	439(31.2)	
Low	16(12.1)	430(33.8)	446(31.7)	
Type of family				0.41
Monogamy	124(93.9)	1217(95.5)	1361(93.2)	
Polygamy	8(6.1)	57(4.5)	65(6.8)	
Form of parenthood				0.003
Both parents	125(94.7)	1061(83.3)	1186(84.4)	
One parent	1(0.8)	46(3.5)	46(3.3)	
Guardian	6(4.5)	174(13.2)	174(12.3)	
Birth rank				0.53
Only child	5(3.8)	33(2.6)	38(2.3)	
First child	26(19.7)	307(24.1)	333(23.7)	
Last child	33(25.0)	277(21.7)	310(22.0)	
Others	68(51.5)	657(51.6)	725(51.6)	
Number of siblings				0.10
0	5(3.8)	33(2.6)	38(2.7)	
1–2	28(21.2)	217(17.0)	245(17.5)	
3–4	60(45.5)	510(40.1)	570(40.5)	
>4	39(29.5)	514(40.3)	553(39.3)	

There was no statistically significant difference in the proportion of study participants who had or had not utilized oral health care services in the past by type of family (*P* = 0.41), birth rank (*P* = 0.53), and the number of siblings (*P* = 0.10). A larger proportion of the study participants (94.7 %) living with both parents utilized the oral health care services when compared with those living with one parent or guardians/relatives (*P* = 0.003).

### Utilization of dental services

At the follow up visit 12 months after the study intervention, only 791(56.3 %) of the 1,406 study participants seen at the initial visit were seen during the follow up visit: 615 (43.7 %) participants were lost to follow up. The reasons for the loss were; change of school (34 %), relocation from study area (51.2 %) and early entrance to secondary school from the 4^th^ to 5^th^ classes in primary school (14.8 %).

Out of the 791 children seen during the follow up visit, 116 (14.7 %) had utilized the oral health care services. Of these, 101(87.1 %) children and parents could give reasons for visiting the oral health care centres. Fifteen (12.9 %) could not give any reason and their parents could not be reached by phone.

**Fig. 1:**
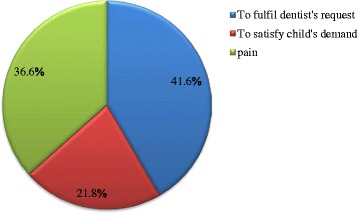
Reasons for utilization of oral health care services

Figure 1 shows the distribution of the 101 study participants who visited the dental clinics to utilize oral health care services. Table 3 shows the distribution of these participants by socio-economic status and family structure. ‘To fulfil the dentist’s request’ (41.6 %) was the reason given by the vast majority who made the dental visit. Majority (72.7 %) of study participants from high socio-economic stratum visited the dentists because the caregivers wanted to satisfy the child’s interest in visiting the dental clinic. None of the parents of participants from the low socio-economic class satisfied their children’s demands to utilize the oral health care service. There was no statistically significant difference observed in the reason for dental visits based on socio-economic status (*p* = 0.22), birth rank (*p* = 0.13) and number of siblings(*p* = 0.28).

**Table 3:** Distribution of study participants who gave reasons for utilization of oral health care services (*N* = 116)

Variable	Pain	To fulfil dentist’s request	To satisfy child	Total	*P* value
	*n* (%)	*n* (%)	*n* (%)	*n* (%)	
Socio-economic status					0.22
High	21(56.8)	27(64.3)	16(72.7)	64(63.3)	
Middle	11(29.7)	10(23.8)	6(27.3)	27(26.7)	
Low	5(13.5)	5(11.9)	0(0.0)	10(10.0)	
Total	37(100.0)	42(100.0)	22(100.0)	101(100.0)	
Type of family					0.19^a^
Monogamy	35(94.6)	42(100.0)	21(95.5)	98(97.0)	
Polygamy	2(5.4))	0(0.0)	1(4.5)	3(3.0)	
Total	37(100.0)	42(100.0)	22(100.0)	101(100.0)	
Form of parenthood					^b^
Both parents	37(100.0)	42(100.0)	22(100.0)	101(100.0)	
One parent	0(0.0)	0(0.0)	0(0.0)	0(0.0)	
Guardian	0(0.0)	0(0.0)	0(0.0)	0(0.0)	
Total	37(100.0)	42(100.0)	22(100.0)	101(100.0)	
Birth rank					0.13^a^
First child	4(10.8)	13(30.9)	6(27.3)	23(22.8)	
Last child	8(21.6)	7(16.7)	6(27.3)	21(20.8)	
Only child	0(0.0)	0(0.0)	1(4.5)	1(1.0)	
Others	25(67.6)	22(52.4)	9(40.9)	56(55.4)	
Total	37(100.0)	42(100.0)	22(100.0)	101(100.0)	
Number of siblings					0.28^a^
0	0(0.0)	0(0.0)	1(4.6)	1(1.00)	
1–2	11(29.7)	11(26.2)	7(31.8)	29(28.7)	
3–4	12(32.4)	22(52.4)	7(31.8)	41(40.6)	
>4	14(37.9)	9(21.4)	7(31.8)	30(29.7)	
Total	37(100.0)	42(100.0)	22(100.0)	101(100.0)	

^a^Likelihood ratio reported because of small cell size

^b^Indeterminate

### Non utilization of dental services

Table 4 highlights the reasons for non-utilization of oral health care services by socio-economic status, type of family, form of parenthood, birth rank, number of siblings. There was a significant difference in the reasons for non-utilization of oral health care services by socio-economic status (*p* = 0.001). A significant proportion of those whose reason for non-utilization of dental services was loss of referral letters, were from the high socio-economic stratum (46.7 %). Most of those who did not utilize dental services because they forgot to give the referral letters to their parents, were from the middle socio-economic stratum (71.4 %) while a significant proportion of those whose parents did not respond to their prompting to make dental visits were from the low socio-economic stratum (68.0 %).

**Table 4:** Distribution of study participants by reasons for non-utilization of oral health care services (*N* = 675)

	Reasons for non-utilization of oral health care centres								
	No Time	Treatment is expensive	Teeth are healthy	Parent did not respond	Lost referral letter	Parent refused	Children forgot to inform parent	Total	*P* value
	*n* (%)	*n* (%)	*n* (%)	*n* (%)	*n* (%)	*n* (%)	*n* (%)	*n* (%)	
Socio-economic status									0.001
High	109(37.3)	49(30.6)	48(31.2)	3(12.0)	7(46.7)	5(22.7)	2(28.6)	223(33.0)	
Middle	105(36.0)	49(30.6)	46(29.9)	5(20.0)	5(33.3)	8(36.4)	5(71.4)	223(33.0)	
Low	78(26.7)	62(38.8)	60(39.0)	17(68.0)	3(20.0)	9(40.9)	0(0.0)	229(34.0)	
Total	292(100.0)	160(100.0)	154(100.0)	25(100.0)	15(100.0)	22(100.0)	7(1.0)	675(100.0)	
Type of family									0.64
Monogamy	282(96.6)	151(94.4)	149(96.8)	24(96.0)	15(100.0)	20(90.9)	7(100.0)	648(96.0)	
Polygamy	10(3.4)	9(5.6)	5(3.2)	1(4.0)	0(0.0)	2(9.1)	0(0.0)	27(4.0)	
Total	292(100.0)	160(100.0)	154(100.0)	25(100.0)	15(100.0)	22(100.0)	7(100.0)	675(100.0)	
Form of parenthood									0.06
Both parents	256(87.7)	130(81.3)	125(81.2)	18(72.0)	15(100.0)	20(90.9)	7(100.0)	571(84.6)	
One parent	7(2.4)	8(5.0)	7(4.5)	0(0.0)	0(0.0)	1(4.5)	0(0.0)	23(3.4)	
Guardian	29(9.9)	22(13.8)	22(14.3)	7(28.0)	0(0.0)	1(4.5)	0(0.0)	81(12.0)	
Total	292(100.0)	160(100.0)	154(100.0)	25(100.0)	15(100.0)	22(100.0)	7(100.0)	675(100.0)	
Birth rank									0.20
First child	87(29.8)	32(20.0)	29(18.8)	4(16.0)	1(6.7)	6(27.3)	1(14.3)	160(23.7)	
Last child	45(15.4)	44(27.5)	37(24.0)	5(20.0)	4(26.7)	4(18.2)	1(14.3)	140(20.7)	
Only child	6(2.1)	3(1.9)	2(1.3)	0(0.0)	0(0.0)	1(4.5)	0(0.0)	12(1.8)	
Others	154(52.7)	81(50.6)	86(55.8)	16(64.0)	10(66.6)	11(50.0)	5(71.4)	363(53.8)	
Total	292(100.0)	160(100.0)	154(100.0)	25(100.0)	15(100.0)	22(100.0)	7(100.0)	675(100.0)	
Number of siblings									0.67
0	6(2.1)	3(1.9)	2(1.3)	0(0.0)	0(0.0)	1(4.5)	0(0.0)	12(1.8)	
1–2	51(17.5)	21(13.1)	18(11.7)	3(12.0)	0(0.0)	5(22.7)	1(14.3)	99(14.7)	
3–4	129(44.2)	64(40.0)	68(44.2)	11(44.0)	7(46.7)	9(40.9)	4(57.1)	292(43.3)	
>4	106(36.3)	72(45.0)	66(42.9)	11(44.0)	8(53.3)	7(31.8)	2(28.6)	272(40.3)	
Total	292(100.0)	160(100.0)	154(100.0)	25(100.0)	15(100.0)	22(100.0)	7(100.0)	675(100.0)	

There was no statistically significant difference in the reasons given for non-utilization of dental services for type of family (*p* = 0.64), form of parenthood (*p* = 0.06), birth rank (*p* = 0.20) and number of siblings (*p* = 0.67).

### Predisposition to utilization of dental services

Only 40 (5.0 %) of the 791 children had a positive disposition towards dental visits. Table 5 shows there was a significant statistical difference (*p* <0.001) in the proportion of participants who visited the oral health care centres based on their predisposition. Most of the participants (60.0 %) with very positive predisposition utilized the oral health care centres after the study intervention. The proportion of study participants who utilized oral health care centres following the study intervention increased as predisposition towards the centres became more positive.

**Table 5:** Predisposition to visit oral health care centres after issuance of referral letters (*N* = 791)

	Visited oral health care centres after referral			
Predisposition	Yes	No	Total	*P* value
	*n* (%)	*n* (%)	*n* (%)	
				<0.001
Very negative	95(81.9)	640(94.8)	735(92.9)	
Moderately negative	0(0.0)	3(0.4)	3(0.4)	
Negative	3(2.6)	10(1.5)	13(1.6)	
Positive	12(10.3)	18(2.7)	30(3.8)	
Very positive	6 (5.2)	4(5.9)	10(1.3)	
Total	116(100.0)	675(100.0)	791(100.0)	

### Predictors of dental service utilization following study intervention

Table 6 shows the predictors of oral health care service utilization by study participants after the project intervention. The socio-economic status of the child, the type of parenthood and the predisposition of the child to oral health service utilization were significant predictors of dental service utilization. Study participants from the middle (AOR: 0.50; 95 % CI: 0.31–0.79; *P* = 0.003) and low (AOR:0.24; 95 % CI:0.13–0.45; *p* = <0.001) socio-economic strata had decreased odds of utilizing the oral health care services when compared to study participants from the high socio-economic stratum. Those living with guardians/relatives (AOR: 0.08; 95 % CI: 0.01–0.60; *p* = 0.01) also had decreased odds of utilizing the oral health care services when compared to those living with both parents respectively. Study participants with positive perceptions about dental service utilization had increased odd of utilizing the oral health care services when compared with children with negative perceptions about dental service utilization (AOR:2.96; 95 % CI:1.48–5.90; *p* = 0.002).

**Table 6:** Determinants of oral health care services utilization after project intervention (*N* = 126)

Variable	Adjusted OR	95 % CI	*P* value
Socio-economic status			
High	1.00	-	-
Middle	0.50	0.31 – 0.79	0.003
Low	0.24	0.13 – 0.45	<0.001
Form of parenthood			
With both parents	1.00	-	-
With one of the parents	0.00	0.00	0.99
With relative/guardian/step parent	0.08	0.01 – 0.60	0.01
Number of siblings			
No sibling	1.00	-	-
1–2 siblings	1.43	0.41 – 5.02	0.58
3–4 siblings	0.71	0.21– 2.42	0.58
>4 siblings	0.75	0.22 – 2.61	0.65
Predisposition to oral health service utilization			
Negative	1.00	-	-
Positive	2.96	1.48 – 5.90	0.002

## Discussion

The study highlighted that referrals can significantly improve children’s visit to dental clinics. The child’s socio-economic status, form of parenthood and the perception of the child about dental service utilization are significant predictive factors of dental service utilization. This study further highlighted two things – referrals made by dentists are respected as professional judgements and are therefore taken seriously by caregivers, reiterating the importance of referral in school-based oral health programs. Secondly, the influence of the child in purchase decisions - in this case, utilization of oral health care services - is significant and had been highlighted earlier by Mangleburg [30].

The referral letter and the prompting of parents by the child to make a dental visit may have helped parents overcome their inertia for making out time for dental visits for their children. When people are encouraged or motivated to visit the oral health centres, utilization of oral health services improves significantly [31]. Folayan, et al*.* [6] showed the role of dental referral in improving oral health care service utilization among school children in Ile-Ife. The study showed clearly that dental referral made a significant impact on oral health service utilization, increasing uptake of oral health care services. Referrals however were not enough to address all the barriers to dental service utilization for children: the ability of the parents to make out time for dental visits is still a very significant limiting factor for dental service utilization. This is an issue that needs to be explored further to identify how meaningful changes can be made to public dental service delivery to address this barrier to children’s access to oral health care. Currently, the operational time of oral health care centers is the same as office and school hours. Parents and children would therefore have to choose between work and school or dental visits. Where parents and children face these hard choices, the likelihood is that oral health care services would only be utilized when there is a pressing need.

It is also important to consider how to address two significant predictors of dental service utilization - the socio-economic status of the child and the type of parenthood of the child: children living with guardians and children from middle and low socio-economic status where less likely to utilize the dental service following the study intervention. The reduced odds may be due to financial constraints. This had been highlighted in previous studies [16, 17]. Socio-economic status is a composite score of educational level and occupation of the parents. Parents whose educational level is low utilize the dental services less frequently and have low knowledge of dental health [32]. Children of such parents have also been shown to utilize dental services less frequently [33]. This may also be as a result of these parents’ inability to make choices as a result of limited resources [34].

Our finding that children staying with relatives/guardians are less likely to utilize the oral health care services than those staying with both parents is in line with findings in an earlier study in which foster parents were less sensitive to the ill health of foster children, and delayed their treatment, when compared to non-foster children. Referrals were ignored by foster parents [35] similar to our study findings, hereby highlighting that these category of children are vulnerable to dental neglect. Programmes designed to address barrier to oral health care utilization for children will not only need to overcome barriers created by socio-economic status but also reduce the vulnerability of children living with foster parents. Up to 12 % of children in Nigeria live with foster parents [36], a significant figure that cannot be ignored when planning for access to oral health care for children.

The study however had some limitations. First, there was a 43.7 % fall-out rate from the study. This undermined the power of the study. The inability to reach parents of 15 other study participants to obtain information on the reasons for their children’s visit/non-visit to oral health centers further reduced our sample size. Despite these limitations, the study was able to provide very useful information that can help in addressing challenges with access to oral health care for children in the study location. It is recommended that future studies explore the role of school based oral health diagnosis and treatment programs on utilization of oral health care services. This might help to overcome some of the barriers associated with children’s access to oral health care services.

## Conclusion

In conclusion, dental referrals could promote the use of dental services, while parents’ inability to make time for dental visits is a significant factor for non-utilization of oral health care services. The socio-economic status and the type of child’s parenthood are significant predictors of oral health care service utilization by children in the study population. These predictors need to be addressed when packaging programs to enhance increased access of children to dental services, in order to prevent emerging inequality in access to dental service in the study population. Intervention programs may involve providing free dental services for children at schools, thereby enhancing the access of vulnerable children to dental care.

## Abbreviations

DMS: degrees, minutes, seconds
GIS: geographic information system
GPS: global positioning system
LGA: local government area
SPSS: statistical package of social science
